# Malignant transformation of oral leukoplakia: a multicentric retrospective study in Brazilian population

**DOI:** 10.4317/medoral.24175

**Published:** 2020-11-28

**Authors:** João Mateus Mendes Cerqueira, Flávia Sirotheau Corrêa Pontes, Alan Roger Santos-Silva, Oslei Paes de Almeida, Rafael Ferreira e Costa, Felipe Paiva Fonseca, Ricardo Santiago Gomez, Nicolau Conte Neto, Ligia Akiko Ninokata Miyahara, Carla Isabelly Rodrigues-Fernandes, Elieser de Melo Galvão Neto, Anna Luíza Damaceno Araújo, Márcio Ajudarte Lopes, Hélder Antônio Rebelo Pontes

**Affiliations:** 1 Oral Diagnosis Department (Pathology and Semiology), Piracicaba Dental School, University of Campinas, Piracicaba, Brazil; 2 Service of Oral Pathology, João de Barros Barreto University Hospital, Federal University of Pará, Belém, Brazil; 3 Department of Oral Surgery and Pathology, School of Dentistry, Federal University of Minas Gerais, Belo Horizonte, Brazil

## Abstract

**Background:**

Among the oral potentially malignant disorders, leukoplakia stands out as the most prevalent. The purpose of this study was to analyse the clinical-pathological features of oral leukoplakia in groups of patients from three major pathology centers in two different regions of Brazil, in order to determine which factors would be associated to the clinical risk of malignant transformation.

**Material and Methods:**

A total of 148 patients was analyzed, and data regarding gender, age, site, classification of the clinical subtype, harmful habits such as use of tobacco and alcohol, time of evolution and presence of dysplasia were collected. The association between risk factors and malignant transformation was investigated using the chi-square test and Fischer's exact test for correlation of variables. A significance level of 5% (*p*≤0.05) was used.

**Results:**

The mean age of the patients was 60 years, and 56% were female. Most of the lesions (34,5%) were located in the lateral and ventral regions of the tongue. Of the 148 patients, ninety had clinical follow-up. Malignant transformation occurred in 13 patients (8.8%), with an average of 44 months of follow up.

**Conclusions:**

Non-smoker, nonhomogeneous clinical presentation, location at the tongue, and the presence of high degree of dysplasia were statistically relevant factors associated with a higher risk of transformation transformation.

** Key words:**Potentially malignant disorders, leukoplakia, malignant transformation, squamous cell carcinoma.

## Introduction

Oral squamous cell carcinoma (OSCC) is the most common malignancy of the oral cavity, representing 95% of all malignant neoplasms in this anatomical site (1). In spite of the strides which have been made in multimodal therapy, the low survival rates have not significantly improved over the last decades (1,2). Additionally, most patients with OSCC may be affected by several morbidities, including severe functional and cosmetic defects, mucositis, xerostomia and osteoradionecrosis, which impair the patients’ quality of life (3).

Given this complex clinical scenario associated with oral cancer management, the search for effective screening methodologies that aim to identify oral potentially malignant desorders (OPMDs) with higher efficacy and therefore, improve the prognosis of these patients, remains desirable and must be performed. OPMDs represent a group of lesions which carry an increased risk of cancer progression, and most (if not all) OSCCs are preceded by these lesions, particularly oral leukoplakia (OL) (4). OL has a worldwide prevalence of 1-5%, and it is defined by the World Health Organization [2017] as “a white plaque of questionable risk having excluded (other) known diseases or disorders that carry no increased risk for cancer” (5).

A recent meta-analysis of 32 studies presented an estimated overall mean proportion rate of malignant transformation (MT) of 9,3% for OL (6). Other studies have described an annual recurrence rate about 13,5%-17%, following surgical excision (7,8). This is partly due to the study design, variation between ethnic groups, and geographical differences, as well as populations’ local habits (9). Therefore, this study aimed to evaluate the importance of different clinical and microscopic factors for malignant transformation of OL in a Brazilian sample.

## Material and Methods

- Study population

All cases diagnosed as OL between January 2010 and November 2019 were retrospectively retrieved from the pathology files of three Brazilian institutions, as follows: Oral Pathology Service of the João de Barros Barreto University Hospital (Belém); Piracicaba Dental School of the University of Campinas (Piracicaba), and the School of Dentistry of the Universidade Federal de Minas Gerais (Belo Horizonte). All cases were confirmed by microscopic examination following incisional or excisional biopsies.

- Inclusion criteria 

The inclusion criteria comprised cases with clinical presentation of OL, which followed the current guidlines of the World Health Organization Classification of Head and Neck Tumours. Only patients with OL diagnosis and no other concomitant lesions were included in this study.

Demographic and clinicopathological data retrieved included: gender, age, disease location, number ans size of lesions, smoking and/or drinking habit, clinical aspects of the lesion, histopathological diagnosis (including presence of epithelial dysplasia according to binary system), follow-up time (months), status at last follow-up (alive or dead), and time of malignant transformation (months). The patient’s identity remained anonymous according to the Declaration of Helsinki.

According to the size, the lesions were classified as having less or more than 2 cm, as suggested by Speight *et al*. [2018] (9). The patients were divided according to their habits, into 4 groups: current smokers, non-smokers, drinkers, and non-drinkers.

All clinical images were evaluated to confirm the clinical diagnosis of OL and to determine the clinical subtype, as homogeneous or nonhomogeneous (10-11). Homogeneous leukoplakia was characterized by a flat, thin or thick and uniform white plaque with well-defined margins, exhibitting shallow cracks within a smooth, wrinkled or corrugated surface of constant texture. Nonhomogeneous leukoplakia presents different areas of nodular, speckled, granular, and verrucous surface (11). Proliferative verrucous leukoplakia (PVL) is a variant of nonhomogenous leukoplakia, with unknown etiology, which progressively becomes multifocal, and frequently involves the gingiva/alveolar mucosa, buccal mucosa and ventral surface of tongue, as defined by the World Health Organization Classification of Head and Neck Tumours. It is an uncommum and ominous form of OL, with an elevated probability of recurrence after excision, and a high rate of MT. Thus, we separately assessed PVL from nonhomogenous leukoplakia.

- Exclusion criteria

The cases were excluded from our sample according to the following criteria: 1) lack of access to the histological material to allow confirmation of clinical diagnosis; 2) follow-up time of less than 6 months, since a period which encompasses less than 6 months between initial diagnosis and malignancy diagnosis may suggest a simultaneous occurrence of OL and cancer, leading to an overestimated malignant transformation rate of OL (12).

- Histological sample

Formalin-fixed, paraffin-embedded tissues were stained routinely with haematoxylin and eosin (H&E), and analysed by using conventional light microscope. Expert pathologists in the scope of this study without prior knowledge of the clinical data assessed the histological slides to establish histological grades for each case. Oral epithelial dysplasia was classified following the binary grading system, proposed by Kujan *et al*. (13), which labelled the cases as having low risk and/or high risk of malignization.

- Statistical analysis

All gathered data was organized into a database by using the GraphPad Prism (GraphPad Software, In., San Diego, CA), version 8.0. The clinicopathological variables were then submitted to the Chi-square test and Fisher exact test for association. A significance level of 5% (p≤0.05) was adopted.

## Results

- Clinicopathological features

**Figure 1:**
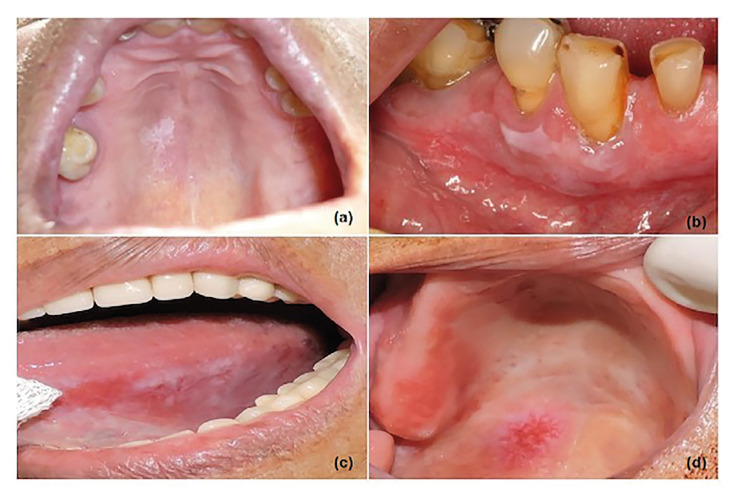
Different clinical presentations of oral leukoplakia. a) A 63-year old male patient, non-smoker, presenting a single lesion on the hard palate, clinically diagnosed as homogeneous leukoplakia. Further microscopic analysis classified the specimen as being of low risk. b) A flat, homogeneous leukoplakia involving the right inferior gingiva of a 78-year old , non smoker female, whose lesion was microscopically considered of low risk. c) A 71 year-old female former smoker exhibiting an extensive lesion with variation of color, on the left border of the tongue, classified as nonhomogeneous leukoplakia. The case was classified as having high risk after microscopic assessment. d) An 82year-old female and non smoker patient, presenting a reddish and white lesion on the palate, which did not show any dysplasia on the microscopic analysis, being classified as low risk.

**Figure 2:**
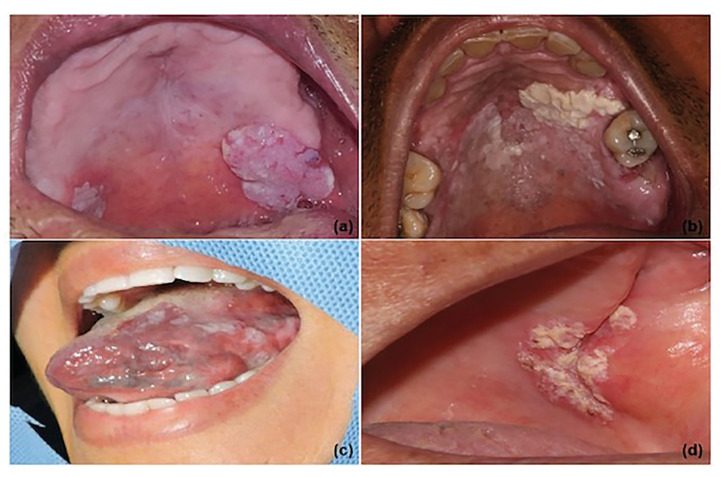
Cases of proliferative verrucous leukoplakia. a) White and reddish lesions of nodular and flat aspects involving the left and right sides of the palate, respectively. The patient is a 75-year old male smoker. The more dense lesion was microscopically classified as high risk. b) A 74-year old male and tobacco user presenting a large lesion, which covered both sides of the hard palate. c) A 31-year old non-smoker female patient, whose prior diagnosis was LVP, was diagnosed with squamous cell carcinoma in the left tongue border after a 71-month follow-up period. d) Another case which progressed to OSCC. A 55-year old male, non-smoker, was initially diagnosed with nonhomogeneous leukoplakia. The malignant transformation occurred after 19 months of follow-up.

From january 2010 to november 2019, a total of 148 patients diagnosed with OL and that fulfilled the required criteria were included in this study. Most of the patients were females (56%), in which the female/male ratio was 1.3/1. The mean age of the patients was 60 years, and most were between 50 to 60 years old. The most involved sites were lateral/ventral surfaces of the tongue (51 cases; 34.5%), followed by multifocal areas (29 cases; 19.6%), palate (21 cases; 14.2%), buccal mucosa (21 cases; 14.2%), alveolar ridge (10 cases; 6.8%), gingiva (9 cases; 6%), and floor of mouth (7 cases; 4.7%). Homogeneous leukoplakia was found in 39.9% [59] of the cases, while nonhomogeneous lesions comprised 38.5% (57 cases). Thirty-two cases were diagnosed as PVL (21.6%). As demonstrated in Table 1, twenty-seven cases had a size of ≤ 2cm, and 121 cases were > 2cm. Fig. 1 illustrates the clinical presentation of different OLs, and Fig. 2 demonstrates 2 cases which progressed to OSCC. From the cases that showed dysplasia at the initial diagnosis, 39.2% (58 cases) were considered as having high risk of malignization. Most patients of the cohort (79 cases; 53.4%) were non-smokers, and 30.4% reported regular alcohol consumption (Table 1).

- Malignant transformation

Ninety patients had available clinical follow-up. A mean follow-up of 36 months was observed. A total of 13 OLs developed OSCC during the follow-up period, resulting in a MT rate of 8.8%, with a mean follow-up of 44 months (Table 1). We found that the progression from OL to malignacy mostly occured in women (8 cases; 61.5%). In addition, the cases who underwent MT comprised patients with an average age of 56 years, ranging from 31 to 76 years at the time of cancer diagnosis. The lateral and ventral surfaces of the tongue were the most common sites for OSCC development. Meanwhile, other tumour sites equally affected the buccal mucosa, gingiva, and alveolar ridge, comprising one case in each location. In relation the PVL, we found only 7.7% of MT rate.

**Figure 3:**
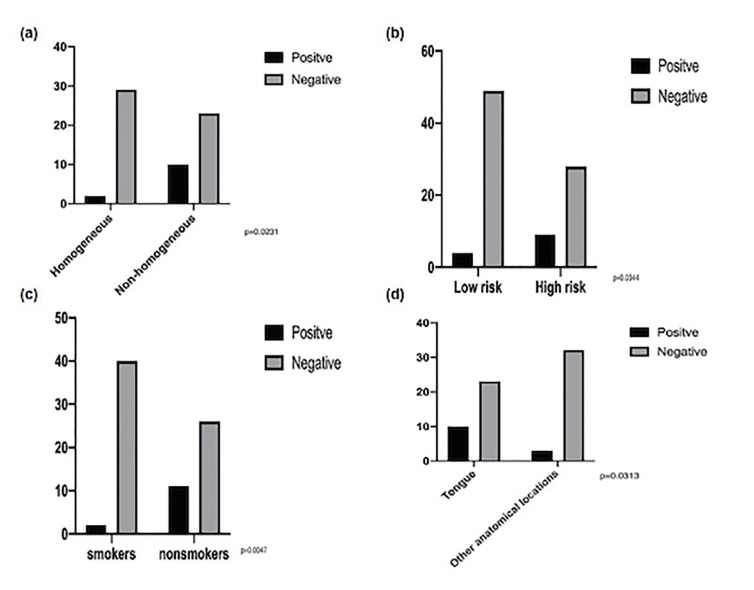
Comparison used the Fisher’s test for the establishment of relations between the malignancy and lesions homogeneous x non homogeneous (a) high risk dysplasia x low risk (b) smokers x non-smokers (c) and location (d).

Statistical analysis showed that the clinicopathological variables which were significantly associated with a higher risk of MT of OL were nonhomogenous lesions (*p* = 0,0231), lesions located on the tongue (*p* = 0,0313), OL with high-grade dysplasia (*p* = 0,0344), and non-smokers patients (*p* = 0,0047) (Fig. 3).

**Table 1:**
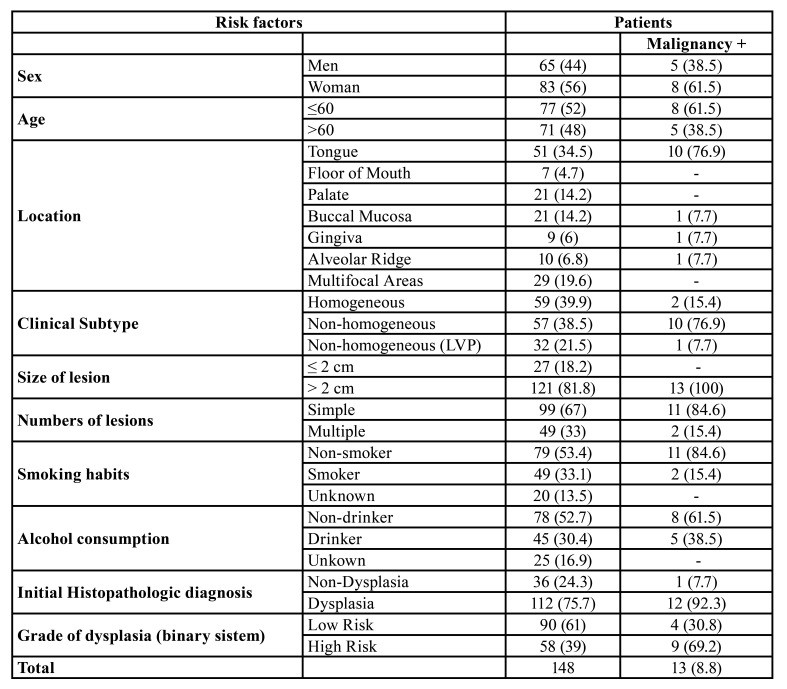
Distribution of risk factors of the 148 patients and malignant transformation rates.

## Discussion

Despite several proposals to characterize the clinical, molecular and microscopic risks of malignant transformation of OL (4,14-16), it is still difficult to predict which type OL will progress to a malignant neoplasm. The MT rate of OL seems to varies among different populations, showing the possible relevance of environmental and host factors (17,18). Although Brazil is a country with the size of a continent, few studies have evaluated the malignant transformation rate of OPMDs, including OL (19).

We found a cumulative MT rate of 8.8% of OL, with an average period of follow-up of 44 months, similarly to the results found by Villa *et al*. [2018] (11) in northen Spain, and the records of a recent meta-analysis, which reported a MT rate of 9.7% (14). In contrast, some studies have shown a MT rate about 12% (7), while other authors demonstrated a MT rate lower than 2% (15,19).

Considering the clinical subtype of OL, our findings are in line with the literature, confirming the high potential of MT of nonhomogenous leukoplakia (11,20,21). Although there is a consensus that PVL has a MT rate higher than 60% (9,11,22), we found a MT rate of only 8% among the cases described as PVL. It is likely that the short follow-up time (36-months) may in part justify our results, since a longer period can be necessary to the evolution of PVL to OSCC. This period may range from 7 to 15 years (22-24), with an average estimated time for MT of 5 to 6 years after diagnosis of PVL (9,25).

It has been stated that histopatholgy alone is not able to provide a MT risk assessment for OL. Besides, oral dysplasia is not considered an indispensable precursor of OSCC, and not all dysplasic OL will transform into cancer (9,15). However, the role of microscopic evaluation cannot be neglected. In fact, our results indicated that the high grade of dysplasia had a significant association with greater risk of malignization, as reported in several studies (6,9,11,26,27). We adopted the binary grading system because it has been considered to present more reproducibility, a best prognostic value, superior reliabiliry, and higher intra/interobserver agreement in comparison with the system proposed by the World Health Organization (9,13).

We also observed a significant higher risk of MT of OL involving the tongue as previously described (10,15,21,27). This is possibly because the cases of tongue OL mostly comprised nonhomogeneous subtypes and demonstrated a high degree of dysplasia. Other possible explanation is related to the high frequency of aneuploidy and loss of heterozygosity of tongue OL, described by several reseachers (28).

Despite the literature has indicates that OL with a size > 200 mm2 presented an increased risk for developing cancer (7,10,18), our results did not show any significant difference between the lesions with > 200 mm2 and < 200 mm2 sizes. This conflicting result may be explained by the fact that the approximately 25% of the lesions > 200 mm2 was PVL, with a limited follow-up period.

Our study also found that smoking was not a significant risk factor for OL malignancy, which is in line with other studies (9,20). Although there are similarities in the genetic alterations found in smokers and non-smokers whose cases suffered MT into oral squamous cell carcinoma (27), previous studies have described a significantly elevated risk for malignant progression in non-smokers (9,27-29), as we observed. Taken together, these data suggest that tobacco plays a crucial role in the formation of keratotic lesions. Subsequently, other factors, which remains unidentified, take the leading role in the progression to malignancy (9,28). In fact, it is unclear why dysplasic OL in non-smokers presents higher risk for MT compared to those that affect smokers.

The major strength of our study was the paticipation of three oral pathology centers from two different regions of Brazil. In contrast, this study has some noteworthy limitations. First, as mentioned above, our sample presented a limited follow-up time for monitoring PVL cases. Second, the group of non-smokers included some ex-smokers with variable periods of habit cessasion, althoug most individuals had stopped smoking 10 years previously. Finally, 58 patients (39,2%) were lost to follow-up over the study period.

In conclusion, non-smokers, nonhomogeneous clinical presentation, location at the tongue, and the presence of high degree of dysplasia were associated with a higher risk of malignant transformation.
